# The Role of Tissue Renin-Angiotensin-Aldosterone System in the Development of Endothelial Dysfunction and Arterial Stiffness

**DOI:** 10.3389/fendo.2013.00161

**Published:** 2013-10-29

**Authors:** Annayya R. Aroor, Vincent G. DeMarco, Guanghong Jia, Zhe Sun, Ravi Nistala, Gerald A. Meininger, James R. Sowers

**Affiliations:** ^1^Department of Internal Medicine, Division of Endocrinology, Diabetes and Metabolism, University of Missouri Columbia School of Medicine, Columbia, MO, USA; ^2^Harry S. Truman Memorial Veterans Hospital, Columbia, MO, USA; ^3^Department of Medical Pharmacology and Physiology, University of Missouri Columbia School of Medicine, Columbia, MO, USA; ^4^Dalton Cardiovascular Research Center, Columbia, MO, USA; ^5^Department of Internal Medicine, Division of Nephrology, University of Missouri Columbia School of Medicine, Columbia, MO, USA

**Keywords:** renin-angiotensin-aldosterone system, arterial stiffness, insulin resistance, endothelial dysfunction, obesity, diabetes

## Abstract

Epidemiological studies support the notion that arterial stiffness is an independent predictor of adverse cardiovascular events contributing significantly to systolic hypertension, impaired ventricular-arterial coupling and diastolic dysfunction, impairment in myocardial oxygen supply and demand, and progression of kidney disease. Although arterial stiffness is associated with aging, it is accelerated in the presence of obesity and diabetes. The prevalence of arterial stiffness parallels the increase of obesity that is occurring in epidemic proportions and is partly driven by a sedentary life style and consumption of a high fructose, high salt, and high fat western diet. Although the underlying mechanisms and mediators of arterial stiffness are not well understood, accumulating evidence supports the role of insulin resistance and endothelial dysfunction. The local tissue renin-angiotensin-aldosterone system (RAAS) in the vascular tissue and immune cells and perivascular adipose tissue is recognized as an important element involved in endothelial dysfunction which contributes significantly to arterial stiffness. Activation of vascular RAAS is seen in humans and animal models of obesity and diabetes, and associated with enhanced oxidative stress and inflammation in the vascular tissue. The cross talk between angiotensin and aldosterone underscores the importance of mineralocorticoid receptors in modulation of insulin resistance, decreased bioavailability of nitric oxide, endothelial dysfunction, and arterial stiffness. In addition, both innate and adaptive immunity are involved in this local tissue activation of RAAS. In this review we will attempt to present a unifying mechanism of how environmental and immunological factors are involved in this local tissue RAAS activation, and the role of this process in the development of endothelial dysfunction and arterial stiffness and targeting tissue RAAS activation.

## Introduction

Arterial stiffness is now considered an independent risk factor for the progression of cardiovascular and chronic kidney disease (CKD) (1). Arterial stiffness increases with aging and is associated with isolated systolic hypertension which occurs in most elderly persons (2). However, the process is accelerated in the presence of obesity and diabetes and occurs at earlier ages (1, 3). Given the association between arterial stiffness and obesity, it is likely that the prevalence of arterial stiffness has been increasing proportionately to the obesity epidemic, which is driven by consumption of a high fat, high fructose, and high salt western diet and further aggravated by a sedentary life style in adults and children in the Unites States and around the globe (4–7). This underscores the importance of arterial stiffness not only as a biomarker for the evaluation of progression of cardiovascular disease (CVD) and kidney disease, but also an important therapeutic target for improved cardiovascular and renal outcomes in obesity and diabetes.

## Arterial Stiffness as a Risk Factor for Cardiovascular and Kidney Disease

Arterial stiffness is associated with obesity, insulin resistance, and activation of the renin-angiotensin-aldosterone system (RAAS) in individuals with the cardiorenal syndrome (CRS) and even in obese children (1, 2, 5, 8). Increased arterial stiffness is also seen in normotensive subjects predisposed to develop hypertension and in pre-hypertensive subjects (9, 10). In the Atherosclerosis Risk in Communities analysis, incident hypertension was more robustly predicted when subjects were in the highest quartile of arterial stiffness. For each standard deviation decrease in elasticity, there was a 15% increase in developing hypertension (11). Arterial stiffness increases with age, metabolic abnormalities, and increased sodium intake, all of which are associated with CVD, including heart failure (12, 13). Furthermore, arterial stiffness itself is associated with left ventricular diastolic dysfunction (14). Increased arterial stiffness is a marker of vasculopathy in CKD patients, suggesting significant cardiovascular damage (15). Arterial stiffness increases with worsening renal function (16). A significant link between aortic pulse wave velocity (PWV) and vascular calcification burden has also been described in CKD patients (17).

## Measurement of Arterial Stiffness: *In vivo, Ex vivo*, and *In vitro*

The evaluation of arterial stiffness *in vivo* in the clinical setting is accomplished by measurement of arterial compliance and distensibility by ultrasound, determination of PWV by measuring the velocity of the pressure wave traveling between two arterial segments, and augmentation index by measuring the augmentation pressure divided by blood pressure (1, 18). PWV closely relates to arterial wall stiffness whereas augmentation index is related to arterial wall stiffness, as well as wave reflection that is dependent on peripheral resistance and affected by heart rate variation (1, 18). The measurement of tissue and cell stiffness *ex vivo* and *in vitro* is greatly enhanced by use of atomic force microscopy (AFM) which can be performed on vascular tissues, endothelial cells, and vascular smooth muscle cells (VSMC) and complimented by confocal imaging (2, 3, 19, 20). Actin can be fluorescently labeled with Alexa 568-phalloidin and cell images, topography, and stiffness recorded with an integrated AFM-confocal microscope system. Furthermore, studies employing AFM probes that have been bio-conjugated with extracellular matrix (ECM) proteins can be used to assess the role of β1-integrin binding and cell adhesion to the ECM. These studies provided a novel concept that both β1-integrin and α-smooth muscle actin play significant role in increased stiffness of VSMCs (2, 3, 20).

## Endothelial Dysfunction, Arterial Stiffness, and Insulin Resistance

### Endothelial dysfunction and arterial stiffness

Arterial intima consists of an endothelial cell layer and underlying layer of smooth muscle cells. It is separated from media by internal elastic lamina. In larger conduit vessels, the medial layer consists of concentric layers of elastic lamina interspersed with collagen and smooth muscle cells (18, 21). The adventitial layer is rich in fibroblasts, macrophages, lymphocytes, adipocytes, dendritic cells, and collagen (22). Arterial stiffness is regulated by a variety of factors including those from endothelial cells, VSMC alterations, cytokines, and inflammatory signals from the adventitia, and characteristic alterations in the ECM. The role of the ECM in modulation of vascular stiffness is well-recognized, and the high elastin to collagen ratio contributes to the elasticity of healthy large arteries (22). With advancing age, there is progressive thickening of arterial walls – predominantly in the intimal layer – with marked increases in the intimal to medial thickness ratio (23). There is also increased fragmentation and depletion of arterial elastin coupled with greater medial deposition of matrix metalloproteins and collagen (18, 21). Collectively, this leads to thicker and stiffer arteries, and is more predominant in the central elastic arteries compared to the peripheral, more muscular arteries. However, the relationships between stiffness in central arteries and more muscular arteries have not been clearly elucidated. The pre-diabetic state is associated with increased arterial stiffness but stiffness was unrelated to vessel wall thickness suggesting mechanisms distinct from ECM remodeling contributing to arterial stiffness (24). In this regard, accumulating evidence suggests a role for the vascular endothelium and provides new insights into the regulation of arterial stiffness (25–27). Endothelial cells regulate several arterial properties including arterial vascular tone and permeability, angiogenesis, and the vascular inflammatory response (25–28). Recently, increased intrinsic stiffness of VSMC has also been implicated in aging (2, 3, 20) and spontaneously hypertensive rats (2, 3, 20, 29). Modulation of transglutaminase 2 (TGM2) by endothelial nitric oxide (NO) (30), identification of vascular smooth muscle cytoskeletal proteins as substrates of TGM2 (31) and inhibition of smooth muscle metalloproteinase expression by NO (32) suggest the role of endothelial and smooth muscle cross talk in modulating arterial stiffness.

### Insulin and RAAS signaling and imbalance of metabolic and growth signaling in the development of endothelial dysfunction and arterial stiffness

**Figure 1:**
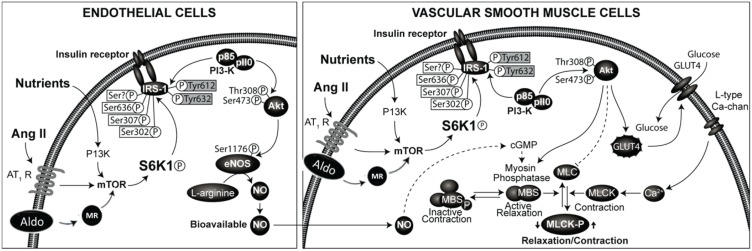
**Renin-angiotensin-aldosterone system and regulation of insulin signaling phosphorylation of docking protein insulin receptor substrate -1 (IRS-1) is the major converging point in insulin signaling**. The phosphorylation of serine residues of IRS-1 by mammalian target of ribosomal p70 S6 kinase (S6K1) acts as a convergence point for the regulation of IRS-1 phosphorylation by nutrients, hormones, and cytokines. Activation of RAAS in endothelial cells and vascular smooth muscle cells leads to inhibition of insulin signaling though phosphorylation of serine residues of IRS-1. This results in impaired signaling though attenuation of phosphatidylinsositol-3 kinase (PI3- kinase)/protein kinase B (Akt) signaling pathway linked to metabolic insulin signaling. This leads to reduced production of nitric oxide and endothelial dysfunction and altered vascular smooth muscle function.

The effects of insulin in the vasculature involve metabolic signaling through the insulin receptor substrate-1 (IRS-1)/phosphatidylinositol 3-kinase (PI3 kinase) AKT/endothelial nitric oxide synthase (eNOS) pathway, as well as growth factor signaling through the ERK1/2/endothelin-1 (ET-1) pathway (28, 33–36). Regulation of endothelial function by insulin metabolic signaling is critical for normal endothelial function and vascular stiffness (1, 8, 33, 34). This insulin metabolic signaling is inhibited by both angiotensin II (Ang II) and aldosterone in vascular endothelial cells and VSMCs (Figure 1). The local vascular effect of insulin beyond systemic effects regulates endothelial activation of eNOS and other signaling pathways (28, 33, 35). In vascular endothelial cells, insulin stimulates production of the vasodilator NO via activation of IRS-1/PI3K signaling (Figure 1) (34, 35). In contrast, growth signaling pathway leads to activation of ERK1/2 and production of the vasoconstrictor ET-1. ET-1 as well as Ang II and aldosterone cause vascular stiffness (1, 8, 28, 36) and increased serum levels of ET-1 are seen in conditions associated with arterial stiffness (36). Activation of the RAAS also leads to impaired IRS-1/PI3K signaling and blunts downstream antioxidant, anti-inflammatory effects of insulin metabolic signaling (22, 34). This, in turn, further impairs insulin-induced vasodilation, capillary recruitment, and augments increases in arterial stiffness (33, 34, 37).

### Insulin resistance, endothelial dysfunction, and arterial stiffness as an early event in progression of CVD and CKD

Endothelial dysfunction is strongly associated with insulin resistance, arterial stiffness, and progression to CVD and CKD (24, 25, 33). Arterial stiffness may also be seen in the absence of insulin resistance in conditions such as hyperglycemia of diabetes mellitus and accumulation of advanced glycation end products (AGE) (37, 38). Individuals with obesity are likely to have an increase in aortic stiffness, independent of blood pressure level. Obesity and arterial stiffness are also independent factors for diastolic dysfunction (38, 39). The occurrence of arterial stiffness and diastolic dysfunction in the absence of elevated blood pressure suggest that arterial stiffness is an early event in the progression to CVD and CKD. In this regard, arterial stiffness is also associated with insulin resistance and an activated RAAS in obesity (38, 39), and insulin resistance alone in the absence of hypertension and coronary heart disease is also associated with diastolic dysfunction (obesity cardiomyopathy) (34). Insulin resistance precedes the development of vascular, cardiac, and renal complications associated with obesity (35). Reduction of aortic dilation to insulin, but not acetylcholine, prior to the onset of hypertension in the spontaneously hypertensive rats (40) and in aged rats (41) provides evidence that insulin resistance is an early event in the development of hypertension.

## Role of Tissue RAAS in Vascular Cells

### Beyond classical and circulating RAAS

Inappropriate activation of RAAS is being increasingly recognized as a major factor in determining endothelial dysfunction, arterial stiffness, and progression to CVD and CKD (37, 38, 42–44). The RAAS is considered as an endocrine system with kidney-derived renin regulating the production of Ang II. In the blood, renin acts on liver-derived angiotensinogen to form Angiotensin I (a decapeptide). Angiotensin I is converted to biologically active Ang II (octapeptide) by the action of endothelial (mainly pulmonary endothelium) derived angiotensin converting enzyme (ACE) (45–49). Ang II acts on adrenals to stimulate the production of aldosterone and on cardiovascular and other tissues to regulate cardiovascular remodeling and blood pressure, in part by inhibiting insulin metabolic signaling in cardiovascular tissues (33, 34, 45) (Figure 1).

In addition to the conventional circulating RAAS, the presence of RAAS components have been detected in tissues such as heart, kidney, vasculature, adipose tissue immune cells, and brain (44–49). Recent studies have shown that VSMCs synthesize angiotensin II intracellularly. Intracellular Ang II regulates the expression of angiotensinogen and renin, generating a feedback loop. The first reaction of intracellular Ang II synthesis is catalyzed by renin or cathepsin D, depending on the cell type, and chymase, not ACE, catalyzes the second step (46, 47). The increased production of Ang II in vascular tissue in conditions of high glucose suggests this component may be of significance in diabetes (46, 47). In additional to the classical Ang II system, the role of non-classical angiotensin peptides generated by tissue ACE2 comprising Ang-(1–9) and Ang-(1–7) which generally antagonize the actions of Ang II are increasingly recognized for their bioactivity (46–49). Ang-(1–7) is also converted to Ang-(1–5) by ACE. Ang III, Ang IV, Ang-(3–7) are other peptides formed from Ang II (46–49). The role for these peptides in vascular tissue is not well understood.

Although the precise role of aldosterone-induced vascular insulin resistance has not been fully elucidated, improved endothelial function in various disease models following treatment with mineralocorticoid receptor (MR) antagonists has been reported (34, 50–53). Blockade of MR by spironolactone decreases local inflammation and vascular stiffness in rodent models of hypertension and insulin resistance (50, 52–54). The contribution of MR signaling to insulin resistance is also supported by insulin resistance in patients with primary hyperaldosteronism (55) and correlation of plasma aldosterone levels with BMI and insulin resistance in normotensive subjects (56).

### Cellular and molecular mechanisms of vascular RAAS-induced insulin resistance, endothelial dysfunction, and arterial stiffness

Molecular mechanisms underlying RAAS-mediated endothelial dysfunction and arterial stiffness in aging, obesity, CRS, and diabetes is not well understood. The role of increased serine phosphorylation of IRS-1 in Ang II and aldosterone-mediated impaired insulin signaling has been demonstrated (33, 34, 57) but the role of mammalian target of rapamycin (mTOR)/S6 kinase (S6K) mediated IRS-1 serine phosphorylation in endothelial cells are not well characterized. We have recently examined the signaling pathways mediating insulin resistance by enhanced activation of tissue RAAS in cardiovascular tissue (57). The serine phosphorylation of IRS-1 was increased and insulin-stimulated phosphorylation of eNOS was decreased by Ang II treatment. Moreover, rapamycin, an inhibitor of (mTOR) activation attenuated Ang II-stimulated phosphorylation of p70S6K and IRS-1 and blocked the ability of Ang II to impair insulin-stimulated phosphorylation of eNOS and NO-dependent arteriole vasodilation. These results suggest the role for activation of mTOR/p70S6K by Ang II in vascular endothelium in mediating impairment of insulin-stimulated vasodilation through phosphorylation of IRS-1 (57). However, MR-dependent effects on endothelial insulin signaling have not been examined.

The role of cross talk between Ang II and aldosterone signaling is increasingly recognized in the development of insulin resistance, endothelial dysfunction, and arterial stiffness (35, 50, 58–60) (Figure 1) and MR blockade attenuates Ang II-induced vascular damage (35, 50, 58, 59). Aldosterone activates NADPH oxidase, thereby promoting oxidative stress and decreased NO bioavailability (34, 50, 61). This is further supported by decreased reactive oxygen species production and agonist-mediated vasoconstriction by specific deletion of VSMC MR in aged mice (59). Aldosterone-induced MR activation increases expression of the intracellular cell adhesion molecule 1 (ICAM-1) (34). Moreover, aldosterone was shown to increase epithelial Na+ channel expression on the endothelial cell surface that correlated with increased cortical stiffness of the cytoskeleton in endothelial cells (62). Of potential importance is that the increase in endothelial cell stiffness was associated with a reduced release of NO (62), which in turn could impact stiffness of VSMC. These observations suggest that inhibition of MR might be a beneficial therapeutic approach for preventing vascular stiffening.

### Up regulation of local intracrine RAAS in obesity, CRS, and diabetes: Role of maladaptive immune and inflammatory response

Although the significance of local RAAS may not be fully understood, the increased expression of RAAS components in vascular tissues in animal models of obesity (63, 64), and direct modulation of vascular RAAS in the vasculature *in vivo* and *in vitro* by insulin (33, 63), uric acid (65), and estrogens (66), favors the role of vascular RAAS modulating endothelial dysfunction and arterial stiffness. Importantly, these factors also cause dysregulation of immune function and a pro-inflammatory response in the vasculature that contribute to endothelial dysfunction and arterial stiffness associated with the consumption of western diet or increased cardiovascular risk in women in the setting of obesity and diabetes.

### Maladaptive immunity and low grade systemic inflammatory response

Accumulating evidence suggests the association of inappropriate activation of RAAS and maladaptive immune and inflammatory responses in modulating endothelial dysfunction and vascular stiffness in obesity and diabetes (67–71). Increased levels of cytokines in the plasma due mainly to visceral adipocyte dysfunction, may contribute significantly to the activation of RAAS in the vascular tissue (38, 68, 69). Moreover, oxidative stress has been shown to cause increased expression of the angiotensin II type-1 (AT1) receptor (68, 69, 71). Decreased levels of interleukin (IL)-10 and impaired function of T-regulatory cells, result in activation of endothelial NADPH oxidase (68, 69, 71). Therefore, an inappropriate activation of RAAS causes cytokine imbalance in plasma and inappropriate activation of RAAS in vascular tissues by cytokines results in a feed forward loop of persistent activation of vascular RAAS in obesity and diabetes (68, 69, 72).

### Perivascular adipocyte dysfunction

The role of perivascular adipose tissue contributing to inflammation, insulin resistance, endothelial dysfunction, and vascular stiffness is increasingly recognized (69, 72–74). In lean mice, perivascular fat exerts protective vasoregulatory effects, but this protective effect is lost in obese mice (74). Endothelial dysfunction in obesity is associated with a significant infiltration of macrophages and T cells in perivascular adipose tissue (72–74). Moreover, perivascular adipose tissue is also a source of Ang II and increased production of Ang II by perivascular fat may also account for impairment of vascular function (75).

### High fructose diet, uric acid, and vascular RAAS

Elevated serum uric acid level is a frequent finding in persons with obesity, hypertension, cardiovascular, and kidney disease. Increased consumption of a fructose-rich western diet also results in elevations in uric acid (6, 7). Elevated serum levels of uric acid appear to contribute to maladaptive immune and inflammatory responses (65, 69, 76), activation of angiotensin system in the vascular cells (65), impaired NO production/endothelial dysfunction (77), and increased vascular stiffness (78, 79).

### Sex differences: Abrogation of cardiovascular protective effects of estradiol in obesity and diabetes in premenopausal women

Females of reproductive age have fewer cardiovascular events however this protection is lost after menopause, suggesting cardio-protective effects of estradiol. The cardio-protective effect of estradiol is also lost in the setting of obesity and diabetes in premenopausal women (69, 80–83). In this regard, arterial stiffness is substantially higher in women than in age-matched men, and is associated with cardiac diastolic dysfunction (82). In a community-based cohort study, increased arterial stiffness was associated with reduced left ventricular diastolic function in both men and women. However, the greater arterial stiffness observed in women was associated with higher incidence of diastolic dysfunction (83–85). Estrogen modulates both Ang II signaling and immune and inflammatory responses. Estradiol normally suppresses actions of Ang II by inhibiting the expression of AT1 (86, 87). However, under the conditions of inhibition of NO synthase and high salt, estradiol increases the expression of AT1 receptor (66, 87). Moreover, GPR-30 which also mediates estradiol effects, increases the expression of ACE2 and decreases the expression of AT1 receptor (88, 89). Estrogen receptor alpha and GPR-30 have been shown to exert an anti-inflammatory effect via modulation of T-cell immune response (90, 91). In addition, estrogen receptor alpha-mediated signaling in macrophages contributes to enhanced insulin sensitivity (92). These findings suggest that a crosstalk between estrogen and Ang II signaling may be one of the factors contributing to sex differences in altered immune and inflammatory responses, endothelial dysfunction, and arterial stiffness, in obesity and diabetes. Furthermore, a recent study demonstrating arterial stiffness in obese pre-menopausal women underscores the role of obesity in abrogating cardiovascular protection in those women (93).

## Conclusion

Arterial stiffness is an independent factor promoting the progression of CVD and renal disease in obesity and diabetes. Inappropriate activation of vascular RAAS in humans and animal models of obesity and diabetes is associated with endothelial dysfunction and arterial stiffness. However, accumulating evidence suggests the role of local tissue RAAS in the vascular tissue, immune cells, and perivascular adipose tissue in endothelial dysfunction contributes significantly to arterial stiffness. The cross talk between angiotensin and aldosterone underscores the importance of the MR in modulation of oxidative stress, insulin resistance, decreased bioavailability of NO, endothelial dysfunction, and arterial stiffness. In addition, both innate and adaptive immunity are involved in local tissue activation of RAAS and in turn are modulated by environmental factors such as high fat/sucrose western diet. Moreover, arterial stiffness is reported in pre-menopausal obese women and estrogen mediated cardiovascular protection is lost in obese or diabetic pre-menopausal women. Taken together, targeting endothelial function and arterial stiffness by modulating tissue RAAS system appears to be an attractive therapeutic strategy to reduce the CVD and CKD complications associated with obesity and diabetes.

## Conflict of Interest Statement

The authors declare that the research was conducted in the absence of any commercial or financial relationships that could be construed as a potential conflict of interest.
